# Collecting Real-Life Psychophysiological Data via Wearables to Better Understand Child Behavior in a Children’s Psychiatric Center: Mixed Methods Study on Feasibility and Implementation

**DOI:** 10.2196/65559

**Published:** 2025-05-30

**Authors:** Karin Hagoort, Kirsten Smeets, Saskia Koldijk, Floortje Scheepers, Fleur Velders

**Affiliations:** 1 Department of Psychiatry, University Medical Center Utrecht, Heidelberglaan 100, Utrecht, 3584 CX, The Netherlands, +31 648463649

**Keywords:** feasibility study, wearable technology, psychophysiological data, arousal, child psychiatry, aggressive behavior, implementation science, wearable, device, clinical care, feasibility, child, children, psychiatric center, psychiatry, mental health, aggression, observational, aggressive, behavior, wristband, psychophysiological arousal, interview, implementation

## Abstract

**Background:**

In the field of mental health care, the incorporation of wearable devices into routine clinical practice continues to face significant challenges, despite the presence of supporting scientific evidence. Crossing the wasteland between the trial world and the real world is full of obstacles that often only become apparent during the implementation process.

**Objective:**

The objective of this paper was to evaluate the feasibility of using wearables in real-world clinical settings for children with severe developmental problems to help understand and manage disruptive behavior and to gain insights for the development of forthcoming implementation strategies.

**Methods:**

A mixed methods design was used to examine two different aspects of the use of wearables in a clinical setting. The first quantitative part of this study focuses on the feasibility of using wearables to collect reliable data on psychophysiological measures during daily activities in children at a children’s psychiatric center. The second qualitative part focuses on the evaluation of the implementation process using the Consolidated Framework for Implementation Research (CFIR) to identify essential steps to successfully incorporate wearable technology in clinical care for children with severe behavioral problems. Empatica E4 wristbands collected data on children’s psychophysiological arousal (eg, heart rate [HR] and skin conductance level [SCL]). Staff reported aggressive behavior and daily activities. Data were processed and visualized in a dashboard. User experiences were assessed through interviews with clinical staff. The implementation process was evaluated using the CFIR.

**Results:**

A total of 30 children (27 boys and 3 girls, aged 6 to 14 y; mean age 9.3 y, SD 1.95) wore the wearable for 5 consecutive days. As expected, the children found it easy to wear the device and the clinical staff predominantly expressed positive attitudes toward its use. The data collection proceeded relatively smoothly, and the collected data were of sufficient quality. In total, 315 observations of aggressive behavior were reported, including 54 red incidents (from 18 unique participants) and 261 orange incidents (from 26 unique participants). An exploratory analysis on the association between psychophysiological measures and aggressive behavior revealed that children’s HR was significantly higher during aggressive incidents compared to nonaggressive incidents (*P*=.007). Although not statistically significant, there was a trend suggesting higher peaks per minute during aggressive incidents (*P*=.07). No significant differences between aggressive and nonaggressive incidents were found for SCL and movement (*P*=.33 and *P*=.60). The most challenging CFIR domains in our study were the “characteristics of the intervention” and “the inner setting,” reflected in the fact that that the majority of implementation activities were focused on these two domains.

**Conclusions:**

The use of wearables in a real-world study setting is considered feasible and valuable. However, for broader scaling in daily clinical practice, coherent actions on different domains of implementation are required.

## Introduction

Technical innovations could be of great benefit in mental health care. Health technology like virtual reality, apps, and wearables have been developed rapidly in highly competitive markets, but only a few are scientifically validated and even fewer ultimately find their way to clinical practice [1]. Wearables have the potential to provide clinicians with valuable and objective real-time data on patients’ physiological and emotional states, both within and outside the clinical setting [2]. These devices enable the measurement of physiological activities, such as electrodermal activity (EDA) and heart rate (HR) [2-5]. This is particularly of interest because elevated psychophysiological measures (ie, HR and EDA) have been linked to emotional and behavioral dysregulation, including aggressive behavior. Given that aggressive behavior is often the main reason of referral for a psychiatric evaluation in a children’s psychiatric clinic and imposes a substantial burden on the child, family, and health care staff, these innovative devices could enhance current treatment programs. By objectively monitoring changes in psychophysiological arousal and subsequent behavior in the patient’s natural environment, we may gain deeper insights into the onset and regulation of aggressive behavior. Although previous studies have explored these effects, research on children and adolescents remains limited [3,4,6-9].

Furthermore, research involving wearables is predominantly conducted in artificial laboratory settings using experimental designs or in small pilot studies. Consequently, there is limited data on the use of wearables in real-time and naturalistic clinical settings [9-11], where they could significantly impact the overall quality of care. Besides the feasibility of collecting psychophysiological data in child psychiatric care with wearables, it is challenging to effectively implement these technologies into real-world clinical practice [12,13]. This gap between inventive technologies and successful implementation processes is commonly referred to as the “valley of death” and creates a waste of research investments [14]. Implementation can be defined as a series of planned and guided activities aimed at introducing and maintaining technologies within a specific context in order to innovate or improve health care [15]. Common obstacles in implementing technology include lack of funding, increased workload for professionals or resistance to using the technology by health care workers or patients in the long term [16]. Hence, the road to sustainable implementation of health technology is a bumpy one and full of obstacles to overcome. However, few studies focused on the process to effectively implement new technologies in clinical practice especially in complex settings like mental health institutes [12,13,17]. To overcome this valley of death and successfully implement innovative technologies, numerous implementation models and frames have been developed [18]. In the Consolidated Framework of Implementation Research (CFIR), common factors from these models have been brought together within one framework aiming to identify potential barriers and facilitators to implementation and guide the development of strategies to optimize implementation outcomes [19,20].

The two aims of our study are (1) to examine the feasibility of using wearables in inpatient clinical care to collect reliable data on psychophysiological measures as a proxy for arousal and (2) to evaluate the implementation process to successfully incorporate wearable technology in clinical care for children with severe behavioral problems.

Taken together, our hypothesis is that it is feasible to use wearable technology (in this case Empatica E4) in clinical care, aiming to provide children and their environment with more insight into their emotional and physical state and behavior. Using a mixed methods design, this study is composed of a quantitative part in which data is collected using wearables and a qualitative part on user experience and the implementation process. Hence, we focus a priori on outcome and implementation, whereby the intervention is studied while also explicitly observing and gathering information on its implementation [15,21], in an attempt to bridge the aforementioned valley of death.

## Methods

### Participants and Setting 

The study was conducted at the children’s psychiatric center of the University Medical Center in Utrecht (UMCU) in The Netherlands, where children aged 6 to 14 years with severe developmental problems are admitted. The center includes an inpatient ward (clinic) for weekday stays, including evenings and nights, and a daycare unit operating from 8 AM to 3 PM on weekdays (daycare). In total, 18 children from the clinic and 12 children from the day care unit were included (see Study Population subsection in the Results section for further details). The majority of the children attended a special education school located adjacent to the treatment center for at least a few hours each day. No inclusion or exclusion criteria were formulated.

### Ethical Considerations 

The ethics approval was reviewed by the Medical Research Ethics Committee at the UMCU, the Netherlands. The study was evaluated as “no research related medical ethical approval needed” since it imposes a minimal additional burden for the patient with no further interruption or change of their regular clinical program. Participants did not receive compensation. Every Empatica E4 watch has a serial number, which was linked to the personal code of the participant. Only 2 researchers received access to the link between both codes to connect observations to the physiological wearable data. All participants and parents were informed about the goal of the study, data storage, privacy, and the anonymization method of the data and they signed written informed consent. All data will be saved for 15 years after the end of the study.

### Design and Procedures

A mixed methods design was used to examine two different aspects of the use of wearables in a clinical setting. The first mainly quantitative part of this study focuses on the feasibility of using wearables to collect reliable data on psychophysiological measures during daily activities in children at a children’s psychiatric center.

The second mainly qualitative part focuses on the evaluation of the implementation process using the CFIR to identify essential steps to successfully incorporate wearable technology in clinical care for children with severe behavioral problems. The Good Reporting of A Mixed Methods Study (GRAMMS) recommendations were used to report this study [22] (see Checklist 1).

During the study, children were asked to wear the Empatica E4 wristwatch on their nondominant wrist for 5 consecutive days. This device measured EDA, HR, movement, and skin temperature (see the Psychophysiological Data subsection for more detailed information on the measurements). The children wore the wristwatch during all normal daily activities, such as school, sports, and treatment programs. At the clinical ward, the children wore the watch from 8 AM to 8 PM, and at the daycare unit, from 8 AM to 3 PM. Over a period of 5 days, the goal was to collect data for 60 hours per child at the clinic and 35 hours per child at the daycare unit.

In addition, behavioral observations with a focus on potential aggressive behavior of the participants were administrated by staff members and 2 independent researchers (see Material and Measurements subsection). At the end of each day, the data of the devices were collected, saved on the computer by the researchers and uploaded into a dashboard. The dashboard with psychophysiological data of 1 day was shown to the clinical staff members to retrospectively evaluate data peaks during the day. After wearing the wearable for 5 consecutive days, children received a diploma and a first insight in the data was shown to their parents. The data were recorded locally on the device. By connecting the wearable to the computer, the accompanying software extracts and synchronizes all recorded data to the Empatica server (as is default). After downloading all data as a zip file containing several .csv files, the data were only locally stored at the UMCU and deleted from the server. All further processing was done locally on UMCU computers. At the end of the study, we asked therapists to participate in an interview for evaluation of the feasibility of using and implementing wearables in daily clinical care. Furthermore, the implementation activities and experiences during the study were carefully registered in meeting notes and a logbook.

### Materials and Measurements 

#### Psychophysiological Data 

Participants were provided with the Empatica E4 smartwatch [23]. This device was investigated and validated in former research [23]. Sensors in this wristband allow real time measuring of EDA, blood volume pulse (on which HR is based), skin temperature, and x, y, z acceleration measurements (on which movement is based). Since the dashboard provided by Empatica did not contain all the information required to properly assess and interpret the data for clinical practice, a customized dashboard was created. First, the data from the Empatica.csv files were plotted: skin temperature, EDA, HR, and inter-beat interval (IBI). Also, the amount of movement and heart rate variability (HRV; calculated from IBI data) were plotted. Artifact and peak detections were performed on the raw EDA data, which resulted in more detailed parameters such as the skin conductance level (SCL) and the number of peaks per minute (PPM). See the study by de Looff et al [24] and Multimedia Appendix 1, for a detailed explanation of the data processing pipeline, outlining the steps from raw Empatica data to the preprocessed data. To interpret measurements in relation to behavior, a panel was created to display text from observations and corresponding marks were added to the SCL plot. Furthermore, to facilitate interpretability, moving averages and reference lines were added to the plots.

#### Child Behavior 

Child behavior was observed using 2 complementary ways. First, clinical staff recorded child observations for diagnostic purposes in the electronic patient file, following standard procedures. Second, all agitated and potentially aggressive behaviors were documented by independent observers (research interns) or staff (clinical staff and teachers), including the timeframe and specific description of the behavior. Aggressive behavior was coded according to the behavior program coding schema (“traffic light model”) as used in the daycare and clinic (see Table 1). “Green” behavior was classified as normal or very good behavior (eg, receiving a compliment for correct behavior). “Orange” behavior included more disruptive actions, while “red” behavior was considered actual physical aggression and severe disruptive behavior. Clinical staff had no access to the children’s smartwatch data during the day and were unaware of their physiological state of arousal while making their observations.

**Table 1.** Traffic light model for behavioral observations.^a^

Color	Behavior	Label
Green	Child follows the rules and behaves in a desired way.	Good behavior
Orange	Swearing, not listening, challenging, arguing, deriding, being agitated, climbing on furniture, and giving the middle finger.	Disruptive behavior
Red	Kicking, hitting, punching, pulling, threatening, pushing, hurting someone, destroying stuff, discriminating, scratching someone else, and spitting.	Physical aggression and severe disruptive behavior

a Aggressive behavior was coded according to the behavior program coding schema (“traffic light model”) as used in the daycare and clinic. “Green” behavior was classified as normal or very good behavior, “Orange” behavior included more disruptive actions, while “red” behavior was considered actual physical aggression or severe disruptive behavior.

#### User Experience and Implementation

Clinical staff were asked to complete an interview after the monitoring period ended. The interview was guided by the Wearable Computer Rating Scale developed by Knight and Baber [25], which is used in other feasibility studies (eg, [26]). To get more insight in the experiences in clinical practice while using the device, questions focused on experiences of the children wearing the device and experiences of the clinical staff regarding the usability of the device in daily practice (what did you like, what did you not like about it, and did the children wear it reliably) were collected (Multimedia Appendix 2). To evaluate the activities during the implementation process, meeting reports, agenda appointments, email exchanges, and action plans were collected in addition to user experiences.

### Data Analysis

#### Psychophysiological Measures and Child Behavior

To explore the association between psychophysiological measures and aggressive behavior, we analyzed psychophysiological data during instances of aggressive behavior in children (red incidents). Furthermore, 5-minute data frames were extracted at the onset of aggressive behavior for this analysis. The recorded time of the aggression incident is the starting point of the 5-minute data frame per incident. To facilitate within-person comparisons, reference data were extracted for each incident from the same participant, at the same time of day, recorded on other weekdays. Reference days were excluded when aggressive behavior was also observed during the reference episode (35/192 18.2%). The following psychophysiological measures were calculated during aggressive episodes and reference episodes: movement that represents the average levels of movement, as well as HR, SCL, and PPM that refer to nonspecific skin responses. Similar to de Looff et al [4], frames with more than 75% artifacts in the EDA signal were deemed unreliable and were excluded from analysis (354/1723, 20.5%). To determine the extent to which psychophysiological measures during aggressive incidents differ from reference episodes, an exploratory paired-sample *t* tests was performed. A total of 4 hypotheses (movement, HR, SCL, and PPM) were tested with a Bonferroni adjusted alpha level of .013 per test (.05/4). More advanced multivariate analysis was beyond the scope of this feasibility study.

#### Implementation Process

**Figure 1.**
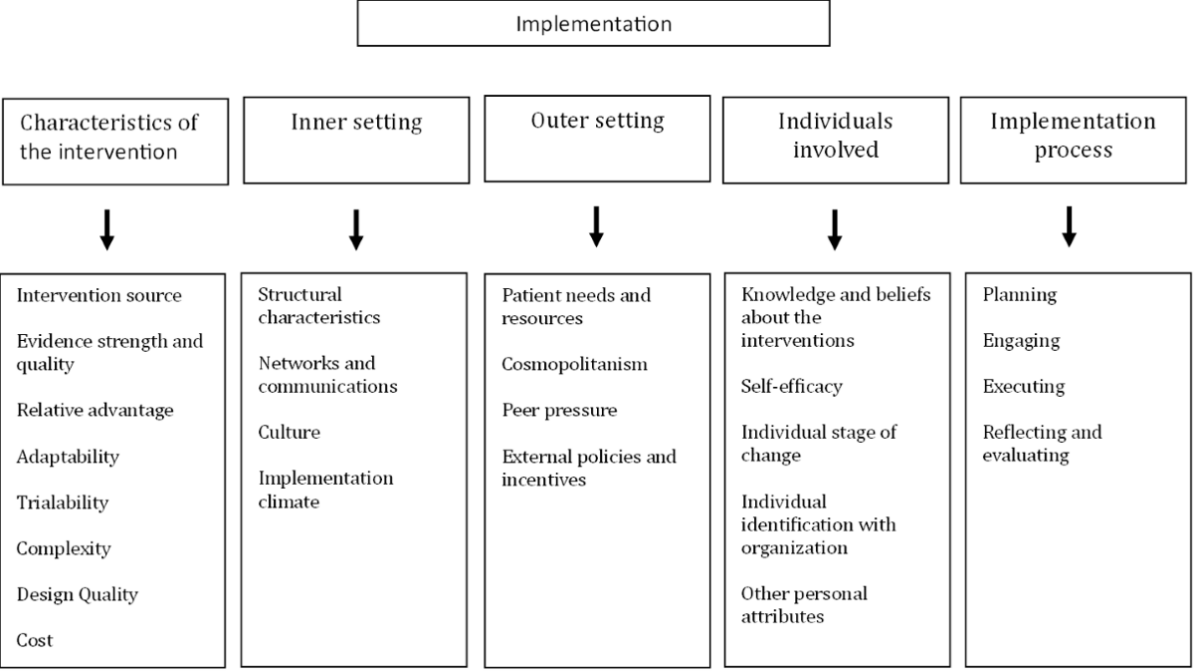
The Consolidated Framework of Implementation Research (CFIR) domains and constructs.

To be able to identify the key barriers and facilitators retrospectively, all implementation activities, events, and incidents from the multiple sources were chronologically listed and categorized with guidance of the 5 domains of the CFIR [18,19]. Each domain includes multiple constructs (see Figure 1) that help evaluate the implementation process and identify potential barriers or facilitators for implementing wearables in clinical practice. Based on the analysis of the activities and the experiences of the stakeholders, the research team discussed and determined which constructs from the different CFIR domains have been relevant to this study and to what extent these constructs were facilitators or barriers. Furthermore, recommendations for future implementation strategies and clinical uptake are given. We describe the findings of the process evaluation, including the identification of facilitators and barriers to implementation within each domain. In addition, we present the lessons learned for further uptake, which strategies facilitated dealing with identified barriers.

## Results

### Study Population

In total, 30 children aged between 6 and 14 years old were included and completed the study. The study population mainly consisted of boys (27/30, 90%), admitted at the clinic (18/30, 60%), and diagnosed with a neurodevelopmental disorder (17/30 56.7%). Table 2 summarizes all descriptive characteristics.

**Table 2.** Demographic and clinical characteristics of pediatric patients from a psychiatric clinic and daycare unit (n=30).

Characteristics	Results
Age (years), mean (SD)	9.3 (1.95)
Sex (male), n (%)	27 (90)
Clinic (vs daycare), n (%)	18 (60)
Primary diagnosis, n (%)	
Neurodevelopmental disorder	
Autism spectrum disorder (ASD)	11 (37)
Attention deficit hyperactivity disorder	5 (17)
Language disorder	1 (3)
Developmental delay	1 (3)
Trauma- and stressor-related disorders	1 (3)
Obsessive compulsive disorder	1 (3)
Disruptive, impulse-control, and conduct disorders	5 (17)
Other conditions	
Parent-child interaction problems	3 (10)
Sibling Relational Problem	1 (3)
Child neglect	1 (3)

### Data Collection

In the clinic, on average, 44 hours of data per child were collected (80% of the aim). In day care treatment, on average, 23 hours of data per child were collected (65% of the aim). Several days show missing data because some of the children did not attend the day care on those days for several reasons. In total, 315 observations of aggressive behavior were reported, including 54 red incidents (from 18 unique participants) and 261 orange incidents (from 26 unique participants).

**Figure 2.**
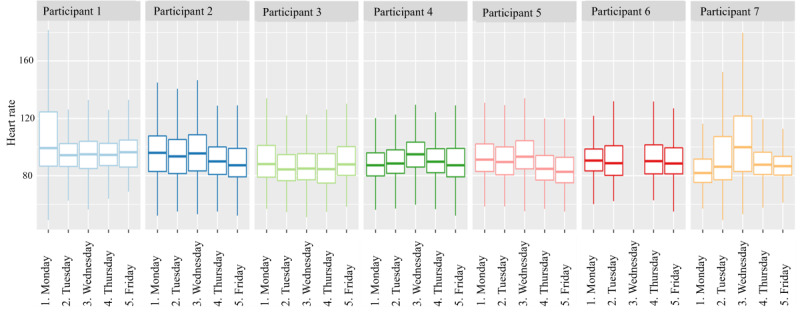
The heart rate (HR) distribution of 7 participants on 5 consecutive days for each patient. On the x-axis time is plotted and on the y-axis the HR in beats per minute is plotted. The distribution is plotted in boxplots, showing the minimum score, first quartile, median, third quartile, and maximum score.

As described earlier, HR is processed automatically. In general, the measured HR frequencies are within the normal range for children of this age, and no missing data were detected. It was noticed, however, that even when the watch is not on the wrist, the Empatica algorithms determine a (artificially high) HR (Multimedia Appendix 3). This explains the exclusion of 3 participants’ outliers. To further assess the reliability of the data, the daily distribution of HR levels for 7 participants was examined. The results showed no significant deviations from the normal range of HR in children, as defined by the Advanced Pediatric Life Support guidelines: 80‐120 bpm for ages 6‐12 years and 60‐100 bpm for ages 12‐14 years (Figure 2).

Besides HR, Empatica also provides IBI, which is the distance between 2 peaks. This is only provided in case 2 consecutive peaks are reliably detected. Only for a small fraction we have valid IBI data, which means that derived measures, like HRV, could not be validly calculated.

For EDA, the Python script “EDA Explorer” was applied to filter the signal ourselves (see Multimedia Appendix 4) and to identify noise. Furthermore, it made visible that most noise is found around the valid signal (see Multimedia Appendix 5). Taking the moving average on the raw signal (as was done in our Dashboard) provides data very similar to the filtered signal. When excluding noise from the EDA data, we distinguish long moments of noise (no valid data for 10 min or the watch was probably not on the wrist) from artifacts in the signal. On average, we collected 39.3 hours of data per participant. After removing long episodes of noise, on average, 34.8 hours of EDA data (−11%) were captured (see Multimedia Appendix 6). After removing artifacts, on average 26.1 hours of EDA data per participant were captured (−25%).

### User Experience

Overall, the children were willing to wear the smartwatch. Only 2 of the 34 recruited children did not want to participate beforehand (while parents gave permission) and 2 parents declined to participate. In Multimedia Appendix 1, a summary of the results from the qualitative data of the clinical staff is presented. They reported that the majority of the children were enthusiastic and felt proud wearing the device. On the other hand, children were critical regarding the convenience of wearing the device. According to the clinical staff, most of the children wore the wearable in a reliable way; some older children were playing with it (turning it on and off or removed it when irritated). They reported unanimously that the behavior of the children and clinical staff was not influenced by wearing the device. With regard to integration in clinical practice, the clinical staff reported that assisting the children with the wearables did not require much additional time and could be easily incorporated into the daily routine. Preferably, the observation of behavior is done by others (ie, research interns), as writing down behavioral observations with the exact time point of the incident or event when also helping the child at the same time is challenging. All clinical staff saw future possibilities of using wearables in clinical practice, mainly in prevention by getting a signal when arousal is rising or use it to get more insight in the arousal of the child. Monitoring sleep was mentioned as a third possible feature. However, ethical considerations were issued as well (“Should we always monitoring the patient?” and “Is it always helpful to know moods and intervene?”). The dashboard was found of additional value to get more insight in what is happening “inside” the child and to use as a starting point to have a conversation especially with children who find it difficult to express themselves. During the study, clinical staff and participants (parents and child) themselves were mostly interested in the visualization of the psychophysiological data on the dashboard. Furthermore, 2 examples are presented below showing the dashboard and its potential use in clinical practice (names have been changed to ensure anonymity).

#### Frank, 10 Years Old

**Figure 3.**
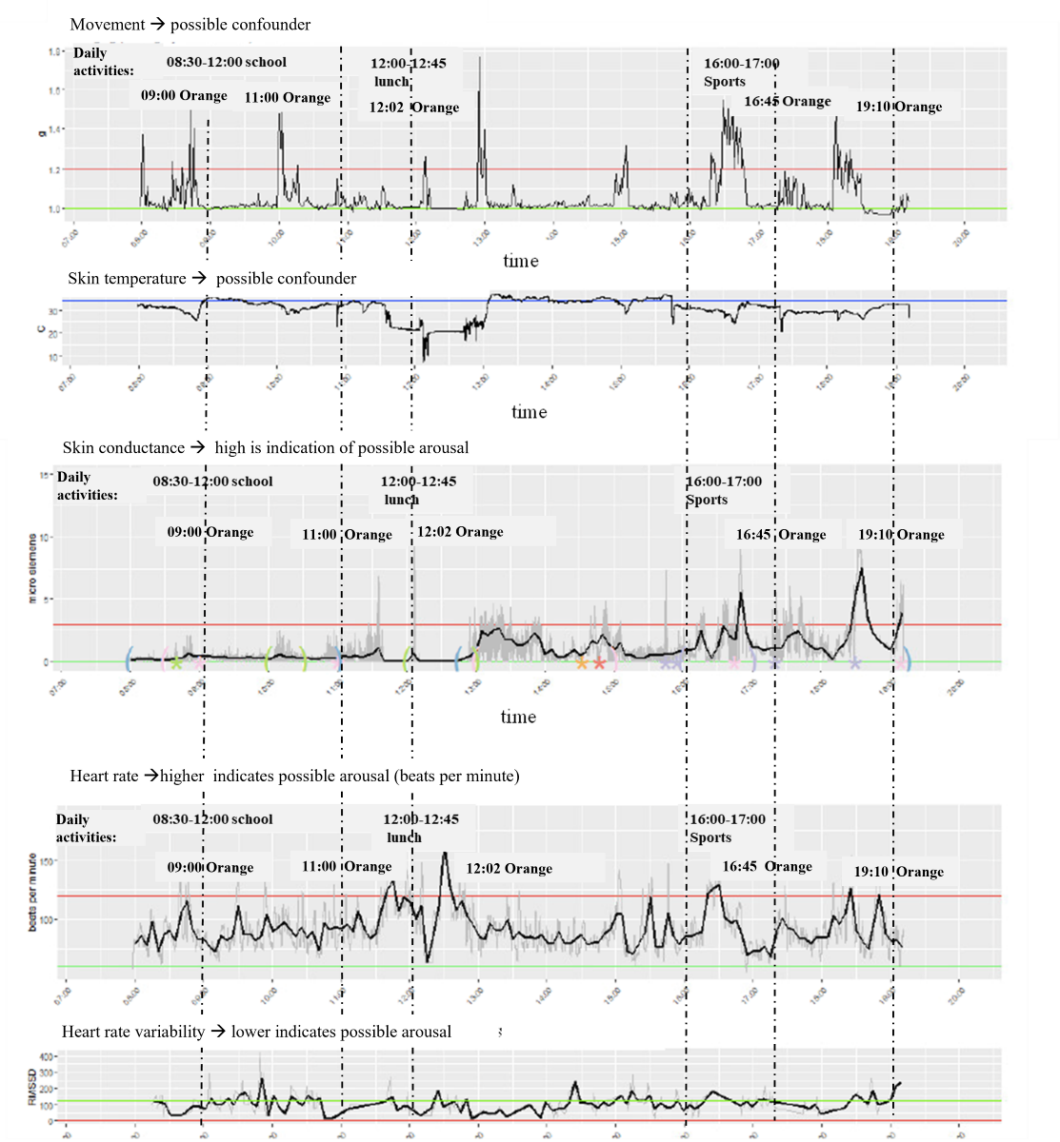
The psychophysiological data of participant X during day 1. On the x-axis, time is plotted. On the y-axis, different psychophysiological parameters are plotted: movement in *g*, skin temperature in Celsius degrees, skin conductance level (SCL) in micro Siemens, heart rate (HR) in beats per minute and heart rate variability (HRV) in root mean square of successive differences. A higher SCL or HR gives an indication of possible arousal, whereas a lower level of HRV indicates a possible level of arousal.

This boy struggled with aggression regulation problems and was previously diagnosed with an autism spectrum disorder. During the data collection week, several red and orange behavioral incidents were reported. Figure 3 shows the data visualization dashboard for the first day when he wore the Empatica E4. Notably, of the 5 orange incidents reported that day, some were associated with a peak in SCL, while others showed no significant change in SCL. The 2 incidents in the morning took place at school. The teacher reported that the boy was removed from class due to misbehavior. Interestingly, no significant peaks in SCL or HR were observed in the dashboard during these 2 incidents. The orange incident around 12:00 noon showed an increase in movement and HR, along with a slight rise in SCL, and occurred while walking from clinic to school. Around 18:30, a substantial peak was observed across different measurements, although no incident or activity was reported at that time.

#### Peter, 12 Years Old

**Figure 4.**
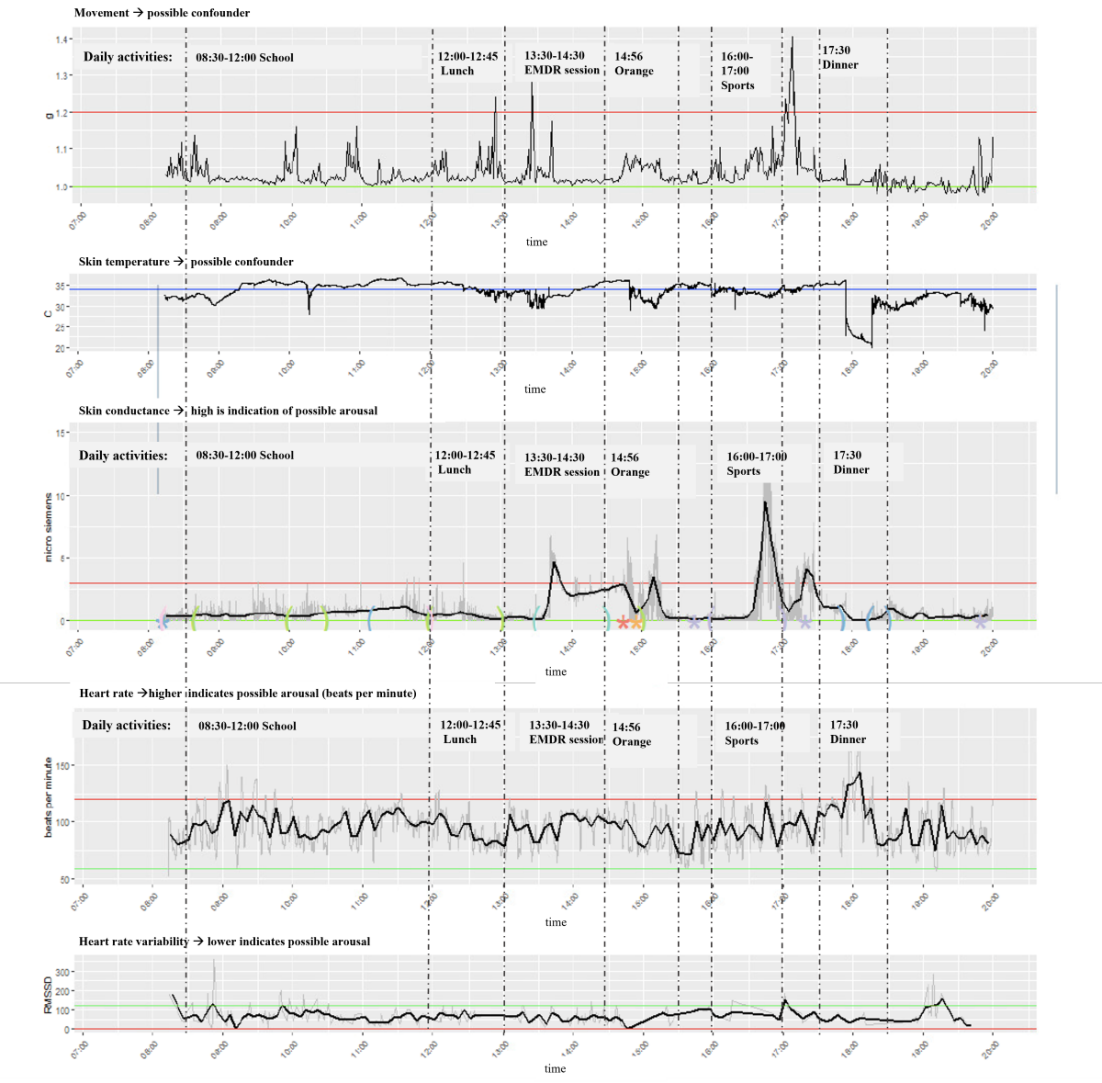
The psychophysiological data of participant Y during day 3. On the x-axis, time is plotted. On the y-axis, different psychophysiological parameters are plotted: movement in *g*, skin temperature in Celsius degrees, skin conductance level (SCL) in micro Siemens, heart rate (HR) in beats per minute and heart rate variability (HRV) in root mean square of successive differences. A higher SCL or HR gives an indication of possible arousal, whereas a lower level of HRV indicates a possible level of arousal.

Peter struggled with several behavioral issues. The main problem was compulsive behavior, and among other things, not speaking and eating; leading to many stressful moments during the day, which could turn into orange or red behavioral incidents (see Methods section). Peter wore the Empatica E4 for 5 days, with the visualization of day 3 presented in Figure 4. Interesting patterns were seen on different timeslots. Peaks in SCL data were observed around the Eye Movement Desensitization and Reprocessing treatment session conducted that day. The therapist reported increased tension during the session. Peter reported that his “subjective unit of distress” decreased from level 7 to level 5 during that session. In addition, an orange incident was reported around 15:00 PM when Peter was informed of a change in his daily routine, requiring him to go to bed early. This change caused significant disappointment and annoyance, potentially explaining the minor SCL peak observed at that time. Furthermore, during physical exercise, an increase in both SCL and movement was noted, underscoring the necessity of accurately interpreting SCL peaks. Such peaks should not be misclassified as stress responses but rather as a result of increased sweating during physical activity. Thus, it is crucial to consider the context and observe behavior and activities during measurements to be able to interpret the data correctly.

### Psychophysiological Measures and Child Aggressive Behavior

An exploratory analysis was performed, in which 4 associations were tested (movement, HR, SCL, and PPM) with paired-sample *t* tests and a Bonferroni adjusted alpha level of .013 per test (.05/4). The HR level of children during aggressive incidents (mean 101.32, SD 19.35) was significantly higher compared to nonaggressive incidents (mean 92.18, SD 8.18; *t*_32_=2.866; *P*=.007). For PPM, the conventional threshold for statistical significance was not reached, but the data may suggest a trend toward a higher PPM during aggressive incidents (mean 3.16, SD 2.00) than during nonaggressive incidents (mean 2.59, SD 1.03; *t*_32_=1.905; *P*=.07). For SCL and movement, no significant difference was found (*P*=.33 and *P*=.60).

### Barriers and Facilitators for Implementation in Clinical Care

**Figure 5.**
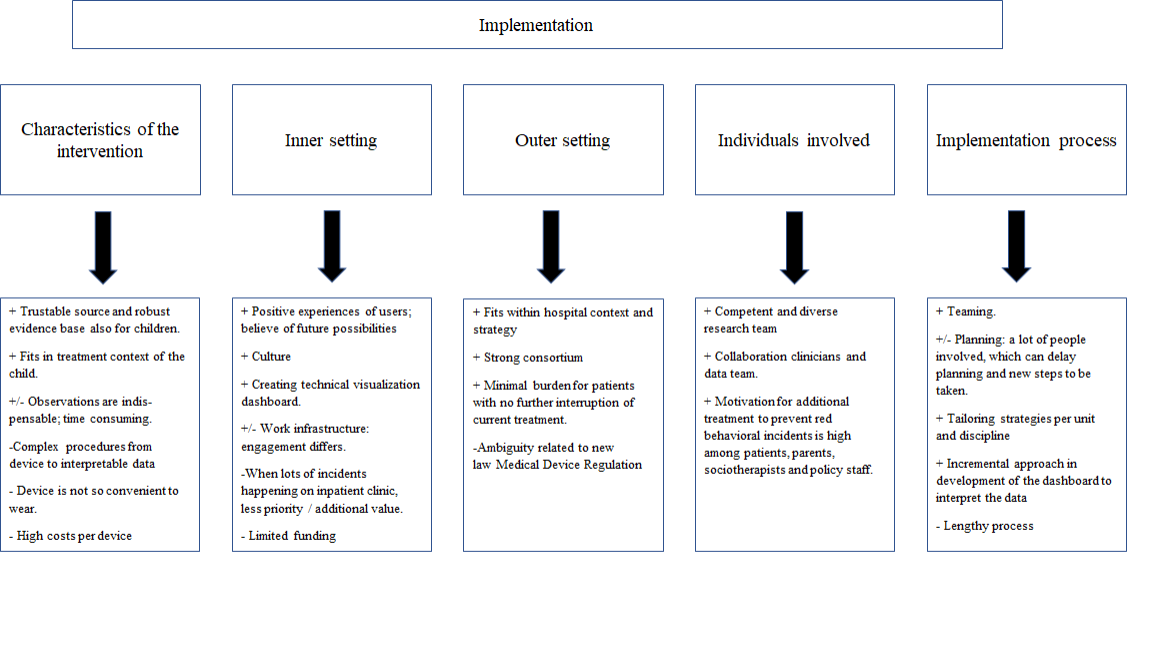
The results of the evaluation of the implementation process using the Consolidated Framework for Implementation Research (CFIR) are presented in terms of factors that had a positive (+), mixed (+/-), or negative (-) impact on the implementation process within each CFIR domain.

The evaluation of the implementation process depicts an intensive and incremental implementation journey to investigate the use of the Empatica E4 in clinical care. Subsequently, key activities and the main facilitating and hindering factors for each CFIR domain were analyzed and discussed below (see Figure 5).

First, the characteristics of the intervention: the intervention encompasses the entire process, involving the wearable device itself, the data collection, and the presentation of data on the dashboard to clinical staff. Positive aspects of the wearable device include its reliability and strong scientific evidence base. User experiences indicate that the devices were easy to operate, although some children reported discomfort while wearing them. The data processing procedure for generating the dashboard was highly customized, with the customization of the dashboard content being perceived as a positive aspect, providing information that was relevant and comprehensible for health care professionals. Simultaneously, the process from data collection to visualization proved to be complex and time-consuming. Downloading the data from the watch, storing, and processing them in the dashboard took approximately 20 minutes per device per day and required the involvement of specific equipment and research staff. For adopting this intervention in daily routine care, the innovation itself should be simplified.

Second, the inner setting: a significant portion of the implementation activities focused on adequately informing, involving, and motivating all stakeholders for participation and cooperation. The extensive coordination required can be attributed to the fact that the study was conducted across two departments, each with its own treatment team, the multidisciplinary nature of the treatment, and the involvement of the school. It was not always evident that information was shared within a team or discipline, often necessitating individual explanations. Throughout the day, many professionals from different disciplines interacted with the children (eg, nurses, psychiatrists, therapists, psychologists, and teachers). Initially, active participation was requested in recording the red, orange, and green incidents, but this proved incompatible with their daily work. This barrier was overcome by deploying dedicated observers during the days the children wore the device. The large number of stakeholders and the necessary coordination with all the different parties stemmed from a culture of high autonomy within disciplines and limited coordination and interdependence. This can also be seen as a barrier within the inner setting that, due to the substantial implementation effort, only minimally hindered the project.

Third, the outer setting: in general, the outer setting can be regarded as a facilitator in this study, as the intervention aligns effectively with the hospital context and its digital strategy. During the study, a new law on Medical Device Regulation was introduced in the European Union. Although the research team could easily approach the legal department in the hospital which is a facilitator, at that moment there was still a lot of uncertainty regarding the impact of the regulation for our study. Due to the inherent ambiguity associated with new legislation, we benefited less from an otherwise facilitating context which causes delay at the start of this study. Furthermore, the research team was integrated into a comprehensive consortium comprising a diverse array of partners with a shared vision that facilitated the provision of wearable devices and contributed to the development of the intervention.

Fourth, the individuals involved: within the domain of “individuals involved” facilitating factors were dominant. The research team which combined the necessary clinical, data science, and implementation expertise, collaborated effectively. It was of significant added value that 2 research team members were also employed at the units of implementation. As a result, they were familiar with the staff, ensuring short communication lines, and enabling them to accurately assess the impact of the innovation and implementation on daily practice. The role of the management of the unit was supportive but in line with the organization culture not decisive. Due to the positive attitude of the majority of the clinical staff, children, and parents toward the innovation, this has not resulted in any hindrance.

Fifth and last, the implementation process: during the study, the teaming was positive, and the absence of a dedicated project leader was compensated for by a clear distribution of tasks. In this project, we offered tailored implementation strategies based on the needs of the units, and due to short feedback loops, we were able to change strategies if needed; for example, the observation that was done first by clinical staff, but since they experienced a lack of time, this was done additionally by research staff in a later phase. The planning could be seen as a barrier. Due to multiple people involved, the 2 different units, and the informal project structure, the time between data collection was not always effectively planned.

In summary, factors emerging from the domain “characteristics of the intervention” and “inner setting” had the most significant influence on the implementation process. Facilitators for implementation are a positive culture for change, motivation of end users, fruitful collaboration of different disciplines, and detailed results of using wearables on qualitative and quantitative levels. Barriers that have to be kept in mind are the type and design of the wearable and the process for data processing, workload, funding, and further research in how to interpret psychophysiological data.

## Discussion

### Principal Findings

This feasibility study examined both the use and implementation of smartwatch-based wearables in a children’s psychiatric center to help children, parents, and staff to better understand children’s behavior.

Using a mixed methods design, the qualitative and quantitative data from this study demonstrate the feasibility of collecting data from this group of children in their natural, yet clinical environment using smartwatch-based wearables. Only by integrating the qualitative and quantitative data, it was possible to thoroughly investigate all aspects of the actual feasibility in clinical care.

With a few exceptions, the children were able to wear the wearable for several days in a row and the data could be collected and processed in an insightful dashboard. No major errors were detected in the psychophysiological data, as collected by the wearables.

Visualizing the data allowed health care professionals to interpret the data and encouraged a more positive attitude toward the use of wearables in clinical care. The results of the single-case explorative visualizations also showed that interpretation of the psychophysiological data is not straightforward and ask for interpretation by the staff based on detailed data on events during the day. In summary, the clinical staff saw opportunities to use psychophysiological data to better understand children’s behavior.

With respect to the second part on the implementation process, this study demonstrates that using wearables in this natural setting required substantial additional effort. The most challenging CFIR domains were “characteristics of the intervention” and “inner setting,” reflected in the fact that that the majority of implementation activities focused on these 2 domains. Considerable effort was invested in designing the intervention to ensure clinical usability and interpretable results. In particular, the data processing from collection to visualization of the psychophysiological data in the dashboard was complex and time-consuming. Hopefully, this process will be automated in the future, to overcome this barrier (eg, [23]). In addition, extensive coordination was necessary with the staff, not only to inform and involve them but also in ensuring the children to wear the wearable. A thorough and customized implementation strategy has ensured successful navigation of these challenges and resulted in a positive attitude toward the innovation. Many of these barriers and facilitators are likely to be generalizable to similar contexts.

### Comparison With Previous Work

Previous research by de Looff et al [4] demonstrated that wearables could be used with adults exhibiting disruptive behavior in a clinical setting. In this study, where we applied the data processing and analysis methods of de Looff et al [4], we showed that it is also possible to measure psychophysiology in hospitalized children with disruptive behavior using wearables.

Even more in line with our study, a small feasibility study (n=10) in a similar group of hospitalized children with disruptive behavior also showed a high adherence rate of using smartwatches in clinical practice [27]. Using other techniques to analyze the data, like the machine learning approach in this study to predict and learn more of the underlying biomarkers of impending disruptive behavior, seems promising and might be the next step to explore in our dataset.

In a scoping review on the use of mobile and wearable artificial intelligence in child and adolescent psychiatry report on 19 articles of which a few also used EDA and HR to predict behavior of children with neurodevelopmental disorders [28]. In addition, 4 out of 19 used similar biosensors and psychophysiological data comparable to our study [9,29-31]. With this study, we address the call in the scoping review of Welch et al [28] for annotated data derived from a naturalistic setting. Our findings also clearly highlighted the challenges and additional effort required to integrate this type of intervention into daily clinical care. This aligns with the challenges mentioned in literature regarding overcoming the gap between the first valley of death and the second valley of death [14]. Bridging the initial gap between evidence-based research in the laboratory and clinical practice is one challenge, but implementing these findings into daily care presents a second gap with additional barriers to overcome.

### Limitations

Several limitations need to be addressed. First, it is challenging to ascertain the clinical relevance of increases in EDA or HR, and to establish baseline levels for individual children, especially for EDA. Second, only for a small fraction do we have valid IBI data, which means that derived measures, such as HRV, could not be validly calculated. This may be due to Empatica’s sensitivity to motion and motion artifacts which affect IBI measurements as reported by Schuurmans et al [32], and that especially affects measurements in young active children. Third, we transitioned from clinical staff to research staff for conducting behavioral observations due to the high time demands. This change did not affect the accuracy of timestamping, indicating that even during the brief period during which clinical staff primarily conducted the observations, they were performed with precision. However, this experience indicates that it will be challenging for clinical teams to maintain this level of time-stamping accuracy as part of their regular daily routine once wearables are integrated into daily practice. Finally, we did not use CFIR in the most rigorous manner, since data collection was not prospectively based on CFIR in qualitative interviews combined with a quantitative survey. This would be of added value but was not feasible in this real-world clinical setting. For this reason, we retrospectively evaluated the implementation process based on project documentation and the experiences of the clinical staff, and the research team identified barriers and facilitators from these sources.

### Conclusions

In line with our hypothesis, the results show that it is feasible to use wearables in a children’s psychiatric center to collect psychophysiological data in children with disruptive behavior. To interpret the data and make it available for clinical staff, numerous steps are required, ranging from data processing to visualizing the data in a dashboard. To help bridge the second gap by actually implementing the use of wearables in daily clinical care, an intervention should require minimal additional time for health care professionals, is intuitive to use, operates without requiring technical support, and is tailored to the clinical context.

Using a dashboard to visualize psychophysiological data and behavioral observations was reported as added value by clinical staff and parents. To optimize the implementation process in other clinical settings, an open access article and code have been made accessible for other researchers and clinicians in the field (eg, [24]). Finally, technical innovations are evolving quickly over time, which is something to keep in mind in the implementation process in the long term (when focusing on one wearable, the other wearable is already in production).

In summary, more research is needed to explore the potential of this innovation to be truly relevant to clinical practice. With these results, we hope to assist organizations in developing an effective tailored implementation strategy for investigating and using wearables in clinical practice, given the evidence that tailored implementation strategies can be effective [33].

Future research should also focus on potential applications for example to gain insight in one’s behavior during therapy, live signaling of increased arousal in different environments, and the added value of virtual reality combined with the feedback by wearables. With the insights from the implementation research, we hope to help accelerate the uptake of such technology in clinical practice.

## Supplementary material

10.2196/65559 Multimedia Appendix 1 Interview questions that were used for qualitative data collection, based on the Wearable Computer Rating Scale and the summary of the results of the interviews.

10.2196/65559 Multimedia Appendix 2 Overview of raw Empatica data, the applied processing and resulting preprocessed dataset.

10.2196/65559 Multimedia Appendix 3 Example of Empatica data as shown in the dashboard. From 16:00 on the watch is not on the wrist, but the Empatica nevertheless provides heart rate (HR measure; measures of skin temperature and electrodermal activity [EDA] do decline and stay at zero).

10.2196/65559 Multimedia Appendix 4 Electrodermal activity (EDA) signal with noise marked in red. Detected by EDA Explorer.

10.2196/65559 Multimedia Appendix 5 Average amount of electrodermal activity (EDA) data per person, from raw to filtered. We distinguish between prolonged noise (>10 min), occurring when the watch was not worn on the wrist, and artifacts in the signal.

10.2196/65559 Multimedia Appendix 6 Electrodermal activity (EDA) signal. First plot: EDA signal including noise; second plot: noise free EDA signal; third plot: moving average of raw EDA signal.

10.2196/65559 Checklist 1 Good Reporting of A Mixed Methods Study (GRAMMS) checklist.

## Abbreviations

ASD: autism spectrum disorder
CFIR: Consolidated Framework of Implementation Research
EDA: electrodermal activity
GRAMMS: Good Reporting of A Mixed Methods Study
HR: heart rate
HRV: hearth rate variability
IBI: inter-beat interval
PPM: peaks per minute
SCL: skin conductance level
UMCU: University Medical Center in Utrecht
