# Supplementary material for: Collecting Real-Life Psychophysiological Data via Wearables to Better Understand Child Behavior in a Children’s Psychiatric Center: Mixed Methods Study on Feasibility and Implementation
Source: JMIR Form Res. 2025 May 30;9:e65559. doi: 10.2196/65559 (PMC12143850; doi:10.2196/65559)
Supplement: Multimedia Appendix 1 [file formative-v9-e65559-s001.docx]

Multimedia appendix 2

**Interview questions which were used for qualitative data collection, based on the Wearable Computer Rating Scale.**

1. What were the experience of the children of wearing the Empatica E4? (i.e. did they like it, did they experience any problems wearing it, were they proud of wearing it)
2. Do you think the children reliably worn the Empatica E4 (i.e. did not turn the device off by themselves)?
3. Did you experience that the children differently behaved while wearing the Empatica E4?

If yes: did it stabilize after a few hours or days wearing the device?

1. Did you experience that the sociotherapists differently behaved towards the childred who were wearing the Empatica E4?

If yes: did it stabilize after a few hours or days wearing the device?

1. What were your experiences with the wearable? What did you like and didn’t like about the Empatica E4?
2. If you could change something of the Empatica E4, what would it be?
3. Did it cost you any extra time, using the wearables in practice? And which method of making behavioral observations worked out the best (i.e. making written registration on a form; someone who is observing the kids whole day etc)?
4. In which way would you use the Empatica E4 in future to support the current clinical health?
5. What were your experiences of using the dashboard (graphics on the laptop) to get an insight in the first data?
6. Do you think the dashboard is of additional value using the wearable?

# Summary of the results of the qualitative structured interviews

|  | **S1** | **S2** | **S3** | **S4** | **S5** | **Conclusion** |
| --- | --- | --- | --- | --- | --- | --- |
| **Q1: Experience of the children?** | Mixed: some didn’t find it comfortable on their wrist; others started to move their wrist a lot (mostly in the beginning). | Mixed: some were fine with it and didn’t notice wearing the device. Others found it less comfortable. | Mixed: most of them liked it and were very proud wearing it. Others found it less comfortable or irritating. | Mixed: most of them liked it; others found it a bit weird and wanted to get more information why wearing it. | Almost all of them liked it very much and were proud wearing the wearables. Some of them forgot to put it back on after showering. | **Mixed experiences; most of the kids liked it but reported that the wearable did not always feel comfortable on their wrist.** |
| **Q2: Did the children wear it in a reliably way?** | It depended on the age. The older children sometimes turned the device off on purpose; the younger children did not. | When starting I had in mind who would possibly turn it off and who wouldn’t; but this was not always right. Only some of the children did that. | Two older children were trying to influence the results of the wearable by turning it off. The others didn’t. I can’t say if it is because of the age of the interaction between those two children. | Mixed: some of them turned the wearable off or removed it from their wrist when getting angry or to see the reaction of the researchers. But most did just wear it. | In my opinion did the children wear the Empatica reliable. | **Mixed experience regarding reliability: most of the children were wearing it reliable, but some of them (mostly the older ones) also played a bit with it or removed it at specific moments.** |
| **Q3: Different behavior of the children while wearing the wearable?** | No | No | No | No | No | **No: the behavior of the children did not change while wearing the Empatica.** |
| **Q4: Different behavior of the sociotherapists while wearing the wearable?** | No | No | No | No | No | **No: the behavior of the sociotherapists did not differ while the children were wearing the Empatica.** |
| **Q5: Experiences of the sociotherapists with the wearable?** | During the research it was easy that we could use 2 wearables per child. The cooperation with researchers who observed the behavior of the child being on school/clinic was pleasant. | No additional information. | Cool device, easy to turn it on and put it on the wrist. | A bit huge for children. | Easy to use. | **User-friendly; helpful when researchers help making behavioral observations; a bit huge.** |
| **Q6: What would you like to change of the wearable?** | It would be nice to have more insight in the data while the children were wearing it and use it also during the night | Children could be even more motivation when they could choose their own color (label) of the wearable. | The wearable is a bit huge for children to wear; it would be more attractive for children if there was some interaction on the wrist: screen which shows a clock, small game, or cartoon that they could choose projected on the watch. | For some of our (sensitive) children the watch was a bit huge and therefore less comfortable on the wrist. Recommendation: Material of the wristband could be more soft and flexible. | Nothing | **Change in the design: less huge for children and interactive screen would be nice. Use of more flexible and softer material.**  **When the children are wearing the Empatica it would be nice to have more insight in the data already.** |
| **Q7: Use in clinical practice (Did it cost you extra time? How to make behavioral observations?)** | Yes, it costs some extra time; mainly because of the behavioral observations. However, during the day the research assistants made observations and we did that during the evening; that worked out very well. | Because of the research assistants who made observations and helped turning on the watches; it barely costed extra time. | It costed some extra time to put the watch on the wrist. It was time consuming that research assistants made behavioral observations. | With the research assistants who made behavioral observations it costed minimal extra time. If we have to make the registrations ourselves it would be too much time and less reliable; since we are too busy with the children and their program on the group. | Yes it costs some extra time; however it was doable because of the research assistants who made the behavioral observations. Only extra time needed to learn how to use the wearable (putting it on the wrist; take it off when the child had to shower etc). | **Using the wearable in clinical practice costs minimal extra time, but external behavioral observations by i.e. research interns is needed.** |
| **Q8: Future support of wearable in clinical practice?** | If possible I would standardly use it in treatment of children with behavioral- and sleep problems. | 1.It would be very interesting if the wearable could be directly connected to the computer, so we get a signal when arousal is rising. But first empirical studies has to be done to prove that it would work like that.  2.Other way would be to check after an incident on which timepoint physiological data was rising; but most of the time we also see that happen. | 1.Would be nice if we could get a signal when i.e. heartbeat is rising so we could help the child with i.e. emotion cards what is happening on that moment. Furthermore, it would be nice to use it to give children more insight in what they feel en when arousal is rising.  2. Use it to detect sleeping problems and reasons why the child is not sleeping well (i.e. stress?) | 1. Would be nice if we could use the wearable more preventive: that we can act earlier; before anger levels get too high and outburst takes place.  On the other hand it feels also a bit weird to constantly measure someone’s state of ‘stress’ and get a signal. It feels as a ethical question. For example, the child is less able to retire to their room i.e. when feeling angry because we can ‘see’ every mood. Maybe it is not always good to know every mood swing and intervene. But sometimes it can help to prevent a big escalation and that would be a positive thing. | It is very helpful for us and the child when a child is less able to express his emotions. The wearable gives insight if there is any stress or not. Using the dashboard is helpful to have a conversation about the behavior and possible stress. | **All sociotherapists see possible future in using wearables in clinical practice, mainly in prevention by getting a signal when arousal is rising or use it to give more insight in their feelings. Monitoring sleep is a third possible feature.** |
| **Q9:Experience of the dashboard?** | Nice and interesting, would have loved to see more data. | Pleasant, when we have time for it. | Very interesting and of additional value; would be helpful to get insight in the dashboard everyday. | Very nice and interesting; gives a lot of information and insight. | Very nice; helpful in understanding if the child is have real stress on a very high level or how to interpret their behavior otherwise. | **Positive experiences were reported of using the dashboard.** |
| **Q10: Additional value of the dashboard?** | Yes, gives more insight in the child and its behavior. | Mixed: nice to see, but not of additional value to our own observations. | Very interesting and of additional value. Mainly in children that do not express themselves very much, we got way more insight in their feelings and stressful situations | Yes, helpful to link it to our own observations and if it matches or not. | Yes, in some of the children and others not. But in one child it was impressive to see that tension building was very high, while we did not see and mentioned that at all with our observations. After that understood more how though it was for him and understood him better. | **Overall, almost all sociotherapists found the dashboard of additional value.** |
