# Supplementary figures and images for: Collecting Real-Life Psychophysiological Data via Wearables to Better Understand Child Behavior in a Children’s Psychiatric Center: Mixed Methods Study on Feasibility and Implementation
Source: JMIR Form Res. 2025 May 30;9:e65559. doi: 10.2196/65559 (PMC12143850; doi:10.2196/65559)

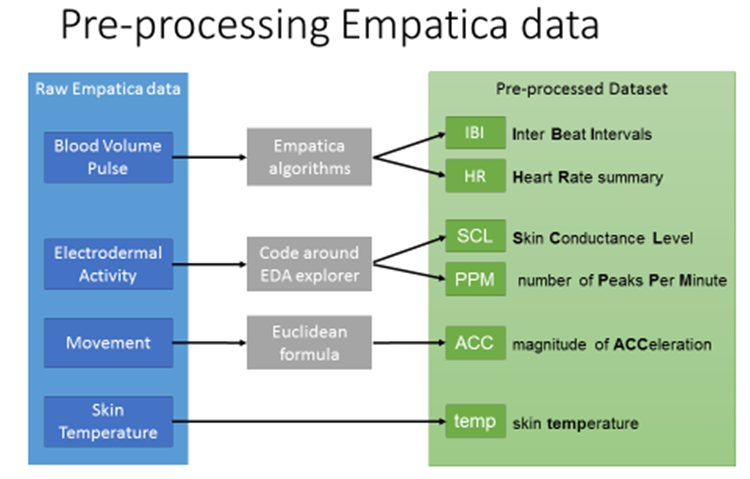

Supplement: Multimedia Appendix 2 [file formative-v9-e65559-s002.png]

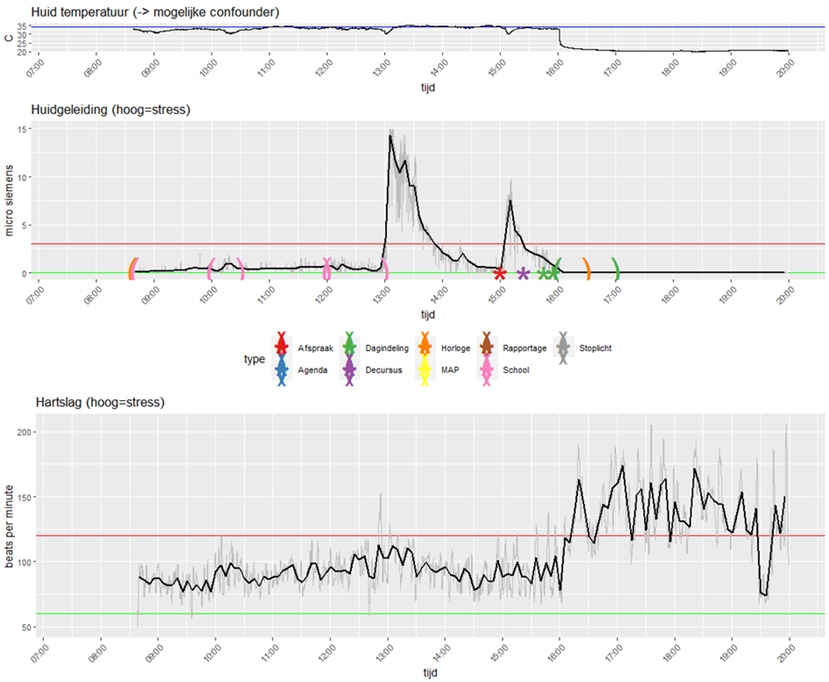

Supplement: Multimedia Appendix 3 [file formative-v9-e65559-s003.png]

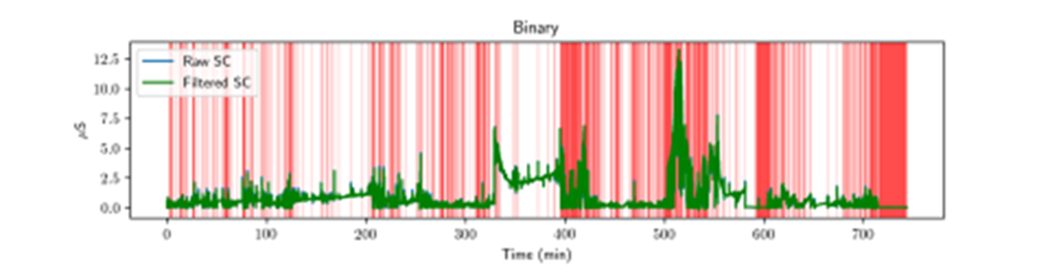

Supplement: Multimedia Appendix 4 [file formative-v9-e65559-s004.png]

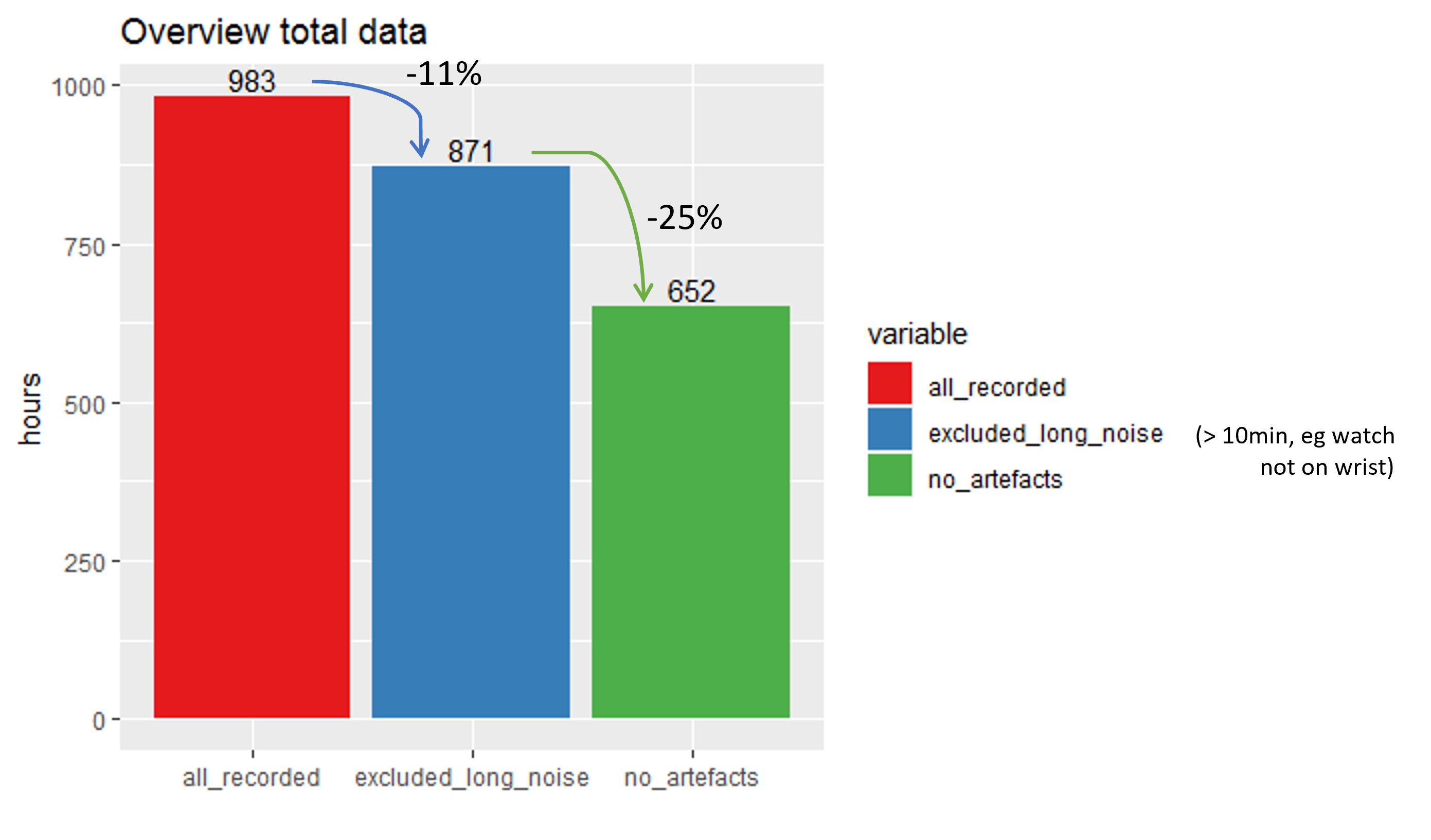

Supplement: Multimedia Appendix 5 [file formative-v9-e65559-s005.png]

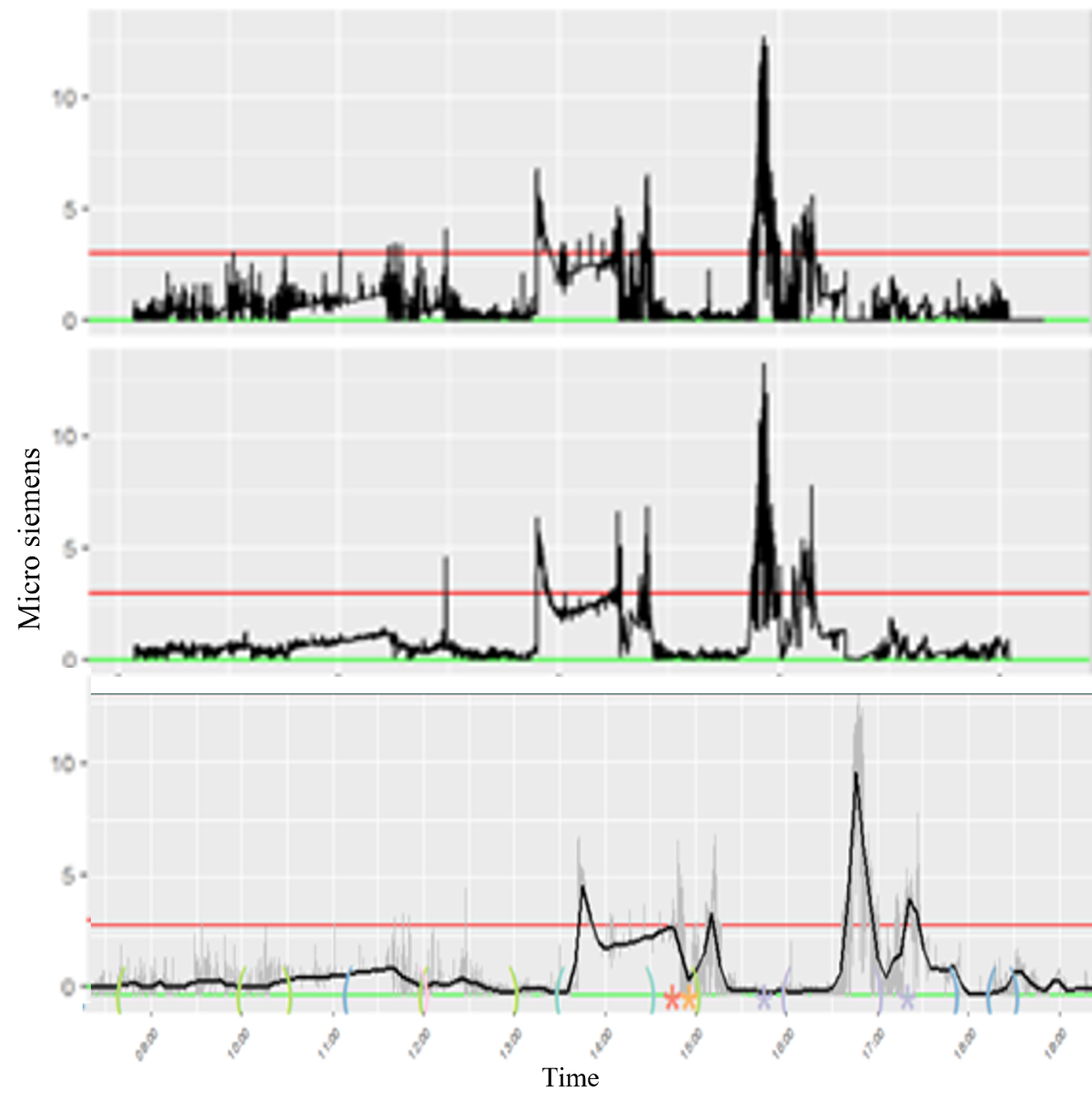

Supplement: Multimedia Appendix 6 [file formative-v9-e65559-s006.png]
