# Supplementary material for: Collecting Real-Life Psychophysiological Data via Wearables to Better Understand Child Behavior in a Children’s Psychiatric Center: Mixed Methods Study on Feasibility and Implementation
Source: JMIR Form Res. 2025 May 30;9:e65559. doi: 10.2196/65559 (PMC12143850; doi:10.2196/65559)
Supplement: Checklist 1 [file formative-v9-e65559-s007.docx]

Good Reporting of A Mixed Methods Study (GRAMMS) guideline.

| 1. Describe the justification for using a mixed methods approach to the research question | See Introduction and Methods, section Design and procedures. |
| --- | --- |
| 2. Describe the design in terms of the purpose, priority and sequence of methods | See Methods, section Design and procedures. |
| 3. Describe each method in terms of sampling, data collection and analysis | See Methods, section Material and measurements and section Data analysis. |
| 4. Describe where integration has occurred, how it has occurred and who has participated in it | See Methods, section Data analysis. |
| 5. Describe any limitation of one method associated with the presence of the other method | No limitations concerning the association between both methods were found. |
| 6. Describe any insights gained from mixing or integrating methods | See Discussion, section Principal findings. |
